# Contribution of Neurotrophins to the Immune System Regulation and Possible Connection to Alcohol Addiction

**DOI:** 10.3390/biology9040063

**Published:** 2020-03-28

**Authors:** Evgenii M. Kozlov, Andrey V. Grechko, Yegor S. Chegodaev, Wei-Kai Wu, Alexander N. Orekhov

**Affiliations:** 1 Laboratory of Clinical Microbiology and Biotechnology of Bacteriophages G.N. Gabrichevsky Moscow Research Institute for Epidemiology and Microbiology, 125212 Moscow, Russia; kozlov-evgeny@bk.ru; 2 Federal Scientific Clinical Center for Resuscitation and Rehabilitation, 109240 Moscow, Russia; noo@fnkcrr.ru; 3 I. M. Sechenov First Moscow State Medical University (Sechenov University), 119146 Moscow, Russia; egozavr-ch@mail.ru; 4 Department of Internal Medicine, National Taiwan University Hospital, Bei-Hu Branch, Taipei 100, Taiwan; weikaiwu0115@gmail.com; 5 Laboratory of Infection Pathology and Molecular Microecology, Institute of Human Morphology, 117418 Moscow, Russia; 6 Laboratory of Angiopathology, Institute of General Pathology and Pathophysiology, 125315 Moscow, Russia

**Keywords:** neurotrophin, alcohol addiction, NGF, BDNF, inflammation

## Abstract

The first references to neurotrophic factors date back to the middle of the 20th century when the nerve growth factor (NGF) was first discovered. Later studies delivered a large amount of data on neurotrophic factors. However, many questions regarding neurotrophin signaling still remain unanswered. One of the principal topics in neurotrophin research is their role in the immune system regulation. Another important research question is the possible involvement of neurotrophin signaling in the pathological processes associated with alcoholism. Among known neurotrophins, NT-4 remains the least studied and appears to be involved in alcoholism and chronic stress pathogenesis. In this review we discuss known neurotrophin signaling cascades mediated by different neurotrophin receptors, as well as provide a generalization of the data regarding the influence of neurotrophins NGF, BDNF, and NT-4 on the immune system and their potential contribution to the pathogenesis of alcoholism.

## 1. Introduction: Neurotrophin Signaling

**Figure 1:**
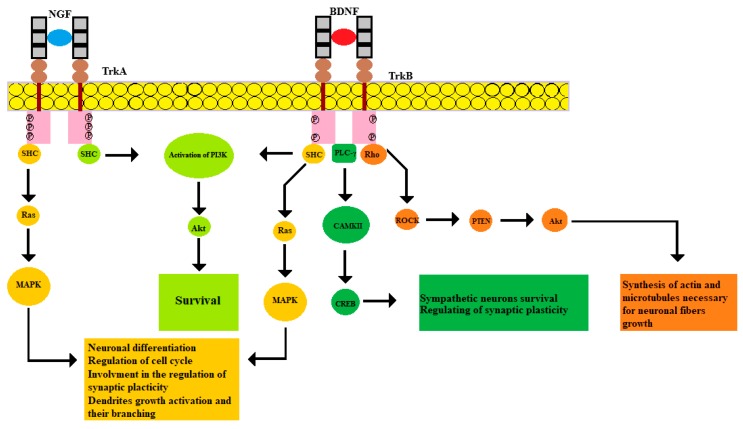
NGF and TrkA binding, just as BDNF and TrkB, leads to dimerization and autophosphorylation of receptors at tyrosine residues. This activates adapter proteins such as SHC, GRB2, GAB1, which ultimately lead to activation of the main signaling pathways. SHC activation involves the Ras-MAPK signaling system, activation of GRB2 and GAB1 mediates signaling along the PI3K-Akt pathway. In addition, phosphorylation of the tyrosine residue 816 TrkB initiates PLC-γ activity, whereby the transcription factor CREB is phosphorylated by CAMKII. Moreover, the signaling that originates from TrkB can activate the Rho family, which leads to the activation of the Rho-associated protein kinase (ROCK), which subsequently leads to activation of the Akt pathway.

Neurotrophins are a family of structurally homologous polypeptides that regulate the survival, development, functioning, and synaptic plasticity of neurons [1,2]. Three subfamilies of neurotrophic factors are distinguished based on their homology of amino acid sequences: neurotrophins (nerve growth factor (NGF), brain-derived neurotrophic factor (BDNF), neurotrophin 3 (NT-3), NT-4, glial factor subfamily (glial cell-derived neurotrophic factor (GDNF), artemin (ART), neurturin (NTR), persephin (PSP)), and ciliary factor subfamily (inhibitory factor (LIF) and interleukin-6 (IL-6)). NGF is a 26 kDa protein, which is synthesized in a form of precursor, a 32 kDa pro-NGF, and packaged into vesicles. Pro-NGF comprises 3 proteins: α-NGF, β-NGF, and γ-NGF. The γ-subunit of this complex acts as a serine protease and cleaves the N-terminus of the β-subunit, resulting in the formation of a mature neurotrophin molecule. NGF is involved in proliferation, differentiation, and survival of sympathetic and sensory neurons, as well as in the regulation of the immune system functions [3,4]. The effects of NGF are mediated through the respective receptors (Figure 1). Tropomyosin receptor kinase A (TrkA) is a high-affinity receptor of NGF, while p75NTR is a low-affinity receptor. However, p75NTR binds pro-NGF with high affinity. This interaction triggers apoptosis and is dependent on sortilin [5]. It was shown that pro-NGF simultaneously binds p75NTR and sortilin to initiate signaling [6]. It should be noted that the formation of this complex was not confirmed by structural biology methods, and the exact signaling mechanism therefore remains to be established [7]. Interestingly, TrkA-mediated NGF activation of ADP-ribosylation factor 6 (ARF6) was shown to be important for increasing the expression of the p75NTR receptor, highlighting the possibility of regulation of sympathetic neurons response to neurotrophins during development [8].

Sortilin is a type I membrane glycoprotein that belongs to the vacuolar protein sorting 10 protein (Vps10p) family of sorting receptors that regulate the intracellular transport by shunting proteins through secretory or endocytic pathways. A study conducted on isolated natural killer (NK) cells demonstrated that pro-NGF could mediate the cell death through activation of the p75NTR-sortin complex. In this study, NK cells were treated with pro-NGF123 or mature NGF. In the presence of IL-12, pro-NGF was shown to mediate apoptosis after 20 h, while without IL-12, this time was 48 h. Moreover, IL-12 promoted the expression of p75NTR, leading to the enhanced apoptotic effect. Blocking the sortilin signaling (pro-NGF, sortilin, and its ligand neurotensin) resulted in attenuation of the apoptotic effect [9].

BDNF is a protein with a molecular weight of 27.8 kDa. Similarly to NGF, it is synthesized as a pro-BDNF precursor by brain glial cells and Schwann cells associated with motor neurons of the spinal cord. The pro-BDNF molecule binds p75NTR and the co-receptor sortilin with high-affinity, inducing apoptosis, long terminal depression (LTD), and synaptic retraction [10].

Historically, pro-BDNF was regarded as an inactive form of the protein, but current research pays greater attention to the precursor forms of neurotrophic factors. One of the important functions of pro-BDNF is to promote apoptosis in neurons (Figure 1) [11]. This function is possibly connected with the removal of damaged neurons or cells lacking functional activity. BDNF activity promotes neuronal survival and increases the amount and differentiation of new neurons and synapses. BDNF functions differ depending on the stage of brain development, as well as on the glial, neuronal, and vascular component of brain tissue [11]. It was shown that pro-BDNF can be anterogradely transported and released from nerve terminations through an unknown mechanism. Pro-BDNF can also form a complex with the Huntingtin associated protein-1 (HAP1) and sortilin, which plays an important role in the transport and stabilization of pro-BDNF [12].

A link between chronic alcohol consumption and disturbance in neurotrophin signaling was established in a study involving 30 men with alcohol addiction and 50 healthy controls. It was found that alcoholism led to disbalance of pro-BDNF–p75NTR–sortilin and mature BDNF (mBDNF)–TrkB interactions. Levels of pro-BDNF, p75NTR, sortilin, mBDNF, TrkB, and their complexes, were measured in the peripheral blood of the study subjects. Concentration of pro-BDNF and p75NTR proteins was increased, while concentration of TrkB reduced in subjects with alcohol addiction in comparison to healthy controls. Levels of mRNA p75NTR and sortilin from lymphocytes were slightly increased, while BDNF and TrkB significantly reduced. The study showed that mBDNF and TrkB levels negatively correlated with average daily ethanol consumption, and pro-BDNF, p75NTR, and sortilin levels positively correlated with the same parameter. Thus, people suffering from alcoholism tend to have a dysregulation of the balance between pro-BDNF/p75NTR/sortilin and mBDNF/TrkB [13,14,15].

**Figure 2:**
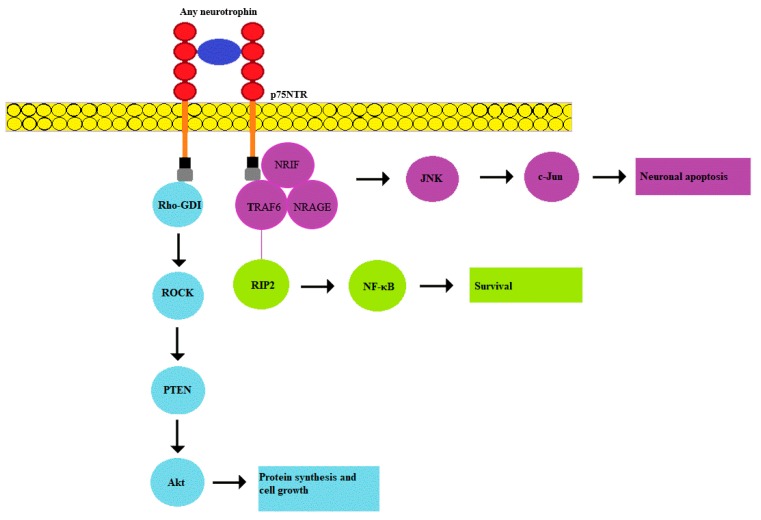
p75NTR is capable of low-affinity binding of any of the neurotrophic factors. The binding of pro-BDNF in combination with sortilin causes the involvement of NRIF, TRAF6, and NRAGE proteins, which activates the JNK-associated pathway. When binding BDNF to p75NTR in the TrkB complex, the RIP2/TRAF6-mediated path is initiated, which leads to NF-KB activation. In addition, p75NTR interacts with the Rho family of proteins, whose activation mediates the activity of Rho-associated protein kinase (ROCK), which subsequently leads to activation of the Akt pathway. RIP2—receptor-interacting serine/threonine-protein kinase 2; NRIF—neurotrophin receptor interacting factor; TRAF6—tumor necrosis factor receptor associated factor 6; NRAGE—neurotrophin receptor-interacting MAGE homologue.

Another member of the neurotrophin family, NT4, is a protein with a molecular weight of 22.4 kDa, which is also synthesized as a precursor. It is involved in the regulation of growth and survival of sensory neurons, motor neurons, as well as dopaminergic and cholinergic neurons. Moreover, it has protective functions against oxidative stress [16]. Interaction of NT4 with TrkB leads to the increase of gene expression and production of factors necessary for neurite growth and neuronal survival [17]. Unlike NGF, NT4 is ubiquitously expressed in the body tissues, but its expression level is low [18]. Similarly to other neurotrophins, NT4 binds a specific TrkB and a non-specific p75NTR receptors (Figure 2) [19]. However, bonding of NT4 to TrkB has distinct effects from BDNF. NT4 elicits long-lasting activation signals, while BDNF signaling is short because of subsequent ubiquitination and degradation of the receptor [17]. Men with severe depression were shown to have a decreased level of NT4 as compared to healthy controls, and this decrease is even more pronounced in women. Deficiency of NT4 may serve as a marker of chronic stress for both women and men with different levels of depression [20].

## 2. Neurotrophin Receptors and Signaling Pathways

### 2.1. NGF Pathway

In humans, 3 subtypes of Trk receptors are known: TrkA, TrkB, and TrkC. The TrkA subtype binds to NGF, the TrkB subtype binds to BDNF and NT4, and the TrkC subtype binds to NT-3. The entire family of neurotrophins can bind to p75NTR. Ligand-receptor interaction leads to conformational changes and the activation of the signaling cascade. Neurotrophins precursors and mature forms are characterized by different effects. According to some results, the activity of pro-NGF is higher than that of the mature form of NGF [21]. At the same time, both NGF and pro-NGF can support cell survival [22]. According to recent studies, the level of TrkA expression plays a key role in controlling the pro-survival signaling. Decreased TrkA expression levels cause the signal switching to apoptotic activity due to the effect of pro-NGF on p75NTR and sortilin [22,23]. Two TrkA subunits are required to activate the TrkB, a high-affinity NGF receptor. NGF and TrkA binding promotes dimerization and subsequent phosphorylation of the cytoplasmic tail by tyrosine residues. Trk phosphorylation sites function as docking sites for the adaptive Src homology 2 domain-containing (Shc) protein. The subsequent activation of the Ras family protein leads to phosphorylation of the Raf protein by the serine/threonine residues, which causes the activation of the mitogen-activated protein kinase (MAPK) cascade. MAPK signaling promotes the transcription of several important factors, including cyclic AMP-response element binding (CREB), which regulates the cell cycle, neurite growth, and synaptic plasticity [24].

The serine/threonine protein kinase Akt is activated via a different mechanism. This pathway begins with the recruitment of the second adapter protein growth factor receptor-bound protein 2 (GRB2) together with the docking protein GRB2-associated-binding protein 1 (GAB1). Then the phosphatidylinositol-3-kinase (PI3K) is activated, which leads to the activation of Akt [25]. This pathway is essential for ensuring cell survival. For instance, Akt signaling inhibits the transcription factors that promote the expression of cell death-related genes and enhance the transcription of antiapoptotic genes [26]. Noteworthy, the Na^+^/Ca^2+^ exchanger of the nuclear envelope of neurons controls the PTEN/Akt signaling pathway through the nucleocytoplasmic ratio of Ca^2+^, therefore regulating neuronal differentiation [27].

Binding of NGF to TrkA, activates transamidase 2 (TG2), which is regulated through the ERK1/2, PKB and PKC-dependent pathway in murine N2a and human SH-SY5Y neuroblastoma cells. A recent study revealed that pharmacological inhibition of TG2 attenuated the NGF-induced growth of neuronal axons. Consequently, it was assumed that TG2 plays an important role in axon growth. Further research is needed to elucidate the mechanisms of work of NGF-induced TG2 [28].

### 2.2. BDNF Pathway

Different isoforms of BDNF interact with distinct receptors mediating different cellular effects. For instance, pro-BDNF interacts with p75NTR and sortilin, while mature BDNF binds to TrkB [29]. The resulting interaction effects of the BDNF isoforms and their receptors are different. For example, the assembly of the pro-BDNF/p75NTR/sortilin complex leads to the activation of c-Jun N-terminal kinases (JNK) and transcription factor NF-κB [30]. Activation of the JNK-associated pathway leads to neuronal apoptosis, while activation of NF-KB, on the contrary, supports neuronal survival [31,32]. Binding of BDNF to the extracellular TrkB domain mediates receptor dimerization and activation of the intracellular tyrosine kinase domain. This leads to tyrosine residues autophosphorylation, which then serve as sites for interaction with adapter proteins and activation of intracellular signaling cascades, including MAPK, PI3K/Akt, PLC, and Rho pathways. Phosphorylation of the tyrosine 515 TrkB residue leads to the involvement of Shc adaptive protein, followed by subsequent recruitment of Grb2 and activation of the Ras-MAPK pathway. The main function of this cascade appears to be the regulation of protein synthesis, dendrites growth activation and their branching [33]. Interaction of Shc-Grb2 involves GAB1 and activates the PI3K/Akt pathway, which provides an anti-apoptotic effect to ensure cell survival [34]. Phosphorylation of the 816 TrkB tyrosine residue leads to PLC-γ attraction, which mediates the hydrolysis of phosphatidylinositol 4,5-bisphosphate (PIP2) to inositol trisphosphate (IP3) and diacylglycerol [35]. Subsequently, IP3 binds to the receptor on the ER promoting Ca^2+^ release, which activates Ca^2+^/calmodulin-dependent protein kinase (CAMK). In its turn, CAMKII phosphorylates CREB transcription factor [36]. Thereby, BDNF can regulate sympathetic neurons survival through CREB-mediated expression of antiapoptotic B Cell Lymphoma-2 (Bcl-2) genes. In addition, it increases the synaptic plasticity [37]. Importantly, the BDNF/TrkB complex initiates GTPase activation, represented by the Rho family, which promotes actin synthesis and microtubules stabilization necessary for neuronal fibers growth [38].

### 2.3. p75NTR Pathway

The p75NTR receptor belongs to the tumor necrosis factor superfamily (TNFR). It has a low affinity for mature neurotrophins, but the entire pool of both precursors and mature forms of these proteins can bind to this receptor with varying strength. p75NTR expression plays an important role during the development of the sympathetic nervous system. Being a pleiotropic signal receptor, it can stimulate survival, growth, apoptosis, and degeneration, depending on the degree of its expression and ligands [39]. Structurally, p75NTR has four cysteine-rich extracellular domains that bind various neurotrophins: NGF, BDNF, and NT-4 with low affinity, as well as corresponding pro-forms that bind with high affinity. The intracellular part of the receptor does not contain a catalytic domain for automatic activation of the receptor unlike Trk receptors [40]. As a result, p75NTR functions in conjunction with other effector proteins. For example, p75NTR can also bind to TrkA to form a high-affinity complex for NGF that promotes neuronal survival, differentiation, and growth. Many aspects of p75NTR activation by neurotrophins are gradually becoming clearer. It was shown that activation of the receptor requires a rearrangement of disulfide-linked receptor subunits [41]. As mentioned above, p75NTR stimulation can lead to cell apoptosis, however, this receptor is usually co-expressed with Trk receptors. Therefore, the survival or death of cells resulting from their activation is determined by the balance of apoptotic and antiapoptotic signals. Overexpression of p75NTR was shown to promote cell death [1]. An interesting quality of p75NTR is its ability to bind not only neurotrophic factors, but also other ligands, such as prion proteins (PrPs) and Aß-peptide amyloid precursor protein (APP) [1]. It was demonstrated that Aβ amyloid peptide regulates the proliferation of neuronal precursors and adult neurogenesis through p75NTR signaling [42].

## 3. Possible Role of Neurotrophins in Alcohol Addiction

The role of neurotrophins in the pathogenesis of alcoholism has been explored by numerous studies. Early studies have shown that chronic consumption of alcohol negatively affected the development of a fetus. Animal studies provided more details on the mechanisms of this influence and revealed the involvement of neurotrophin signaling [43]. A recent study reported that in the offspring of males exposed to alcohol during mating, the expression of NGF/BDNF was altered, potentially causing cognitive decline [44]. Another study revealed the relationship between NGF decrease and cognitive decline in alcohol-dependent patients. Serum NGF concentration was measured in 38 patients with alcohol addiction, and neuropsychological tests were performed to evaluate the state of executive functions. This study found a significant correlation between NGF levels and the Trail-making Test. The increase in NGF concentration was associated with a decrease in the completion time of the hand–eye coordination test [45]. Moreover, available evidence suggests that acute alcohol intoxication is associated with NGF increase, which decreases upon alcohol withdrawal [46]. These results indicate that a high NGF level may have a protective function. Correspondingly, NGF was shown to prevent the death of cortical neurons in a rat cerebral cortex caused by ethanol [47].

The important role of BDNF in the regulation of alcohol consumption has been demonstrated in several studies (Table 1). A correlation was revealed between the risk of alcoholism development and Val68−>Met68 polymorphism in the BDNF gene in mice. The human homologue of the murine Met68BDNF is the Met66BDNF allele [48]. Replacing Val66 with Met66 in the BDNF prodomain significantly altered the intracellular transport of pro-BDNF and reduced the release of this neurotrophin, inhibiting its functions [49,50]. Mice expressing Met68BDNF showed excessive alcohol consumption compared to wild-type Val68BDNF mice. When mice were injected with a TrkB receptor agonist (LM22A-4) or overexpressed Val68BDNF in the ventromedial prefrontal cortex, alcohol consumption decreased. Thus, people carrying the Met66BDNF allele appear to be at a higher risk of developing alcoholism [51].

The role of NT-4 in the development of alcoholism has not yet been clarified and requires further research. One of the mechanisms linking NT-4 signaling with the deleterious effects of alcohol is oxidative stress that has been shown to be associated with chronic alcohol consumption [52]. Ethanol metabolism is known to be linked with the formation of the NADH^+^ reduced form, which is subsequently delivered to mitochondria [53]. There, during the Q-cycle, reactive oxygen species (ROS) formation occurs as a result of an electron leak. Since the concentration of NADH increases due to ethanol metabolism, it may lead to a corresponding increase in ROS production, which may have effect on neurotrophin signaling [54].

## 4. Role of Neurotrophins in the Immune System Regulation

Currently, the contribution of neurotrophic factors to the functioning of both innate and acquired immunity is well studied. It was shown that immune cells (T- and B-lymphocytes, macrophages, and mast cells) express neurotrophins and their receptors [58]. In addition, NGF and BDNF modulate the function of immune cells, acting as mediators of the reciprocal communication between nerve and immune cells [59]. Dysregulation of neuroimmune communication can lead to the development of many diseases that affect both nervous and immune systems, such as Alzheimer’s disease, amyotrophic lateral sclerosis, neuropathy, allergic bronchial asthma, as well as various inflammatory diseases [60,61]. NGF also promotes the survival of hematopoietic stem cells, neutrophils, eosinophils, mast cells, monocytes, and B-cells [55]. Experiments conducted on different cell populations showed that NGF potentiated the inflammatory response by synthesizing cytokines [62]. The development of inflammatory reactions may be associated with p75NTR activation, leading to the activation of the transcription factor NF-κB, which in turn serves as a trigger for the pro-inflammatory cytokines expression. Noteworthy, NGF has a complex effect on the innate immunity system. It modulates monocyte chemotaxis, enhances phagocytosis, induces the expression of pro-inflammatory cytokines by macrophages, and supports neutrophil survival [4].

Interestingly, activated monocytes as well as T and B cells produce BDNF, which may possibly have a neuroprotective effect [62]. After stimulation with cytokines and lipopolysaccharide (LPS), monocytes increase NGF expression, while BDNF expression decreases in the hippocampus and cerebral cortex, which can lead to various nervous system diseases [56,57]. The increase of the pro-inflammatory cytokines level occurs not only through the inflammatory stimuli, but also as an effect of the environmental factors and oxidative stress. For example, it is known that the NF-κB factor is up-regulated during chronic brain stress, leading to the expression of NADPH oxidase (NOX) and ROS generation by glial cells [63]. Oxidative stress, in turn, can have a devastating effect on the integrality of lysosomal membranes, causing the lysosomal hydrolases release, which in the long term leads to inadequate mitophagy. In addition, some mitochondrial mutations suppress mitophagy, causing an increase of pro-inflammatory cascades and chronic inflammation development [64]. Inflammation is known to be a major component of neurodegenerative diseases, and impaired autophagy and, in particular, mitophagy was shown to play a significant role in Alzheimer’s disease [65].

Elevated ROS can have a direct neurotoxic effect, reducing BDNF synthesis and therefore affecting BDNF-dependent neuronal survival. Alcohol-induced oxidative stress induces selective damage of neurons, which leads to apoptosis and neurodegeneration. Ethanol exposure was shown to affect BDNF expression in the hippocampus, brain-cortex, and striatum [66]. Studies on rats assessed the nociceptive response assessed in “tail-flick” and “hot plate” tests and evaluated the levels of BDNF and IL-10 in the prefrontal cortex, brain stem, and hippocampus after alcohol withdrawal. Rats were divided into three groups: a control group, a group that was injected with water, and a group that was injected with alcohol. After 11 days of alcohol withdrawal, a significant BDNF increase was detected in the alcohol-administered group. In addition, IL-10 levels in the hippocampus, prefrontal cortex, and brain stem significantly increased. Thus, prolonged alcohol withdrawal led to an analgesic effect [67]. Analgesia is believed to be associated with increased BDNF and IL-10 levels. Early studies have shown that direct BDNF injection into the midbrain, near the area of periaqueductal gray, causes an analgesic effect, as well as a delay in the “tail-flick” test [68]. BDNF reduced reactions to the exposure to both thermal and chemical stimuli [69]. The mechanisms of such analgesia are not fully understood and require more in-depth study. Moreover, chronic alcohol consumption reduces the number of peripheral T cells, disrupts the balance between their different types, and promotes their apoptosis. Besides chronic alcohol exposure is associated with loss of peripheral B-cells, mainly B-2 lymphocytes [70]. Such a loss explains the compromised response of patients with chronic alcohol consumption to new antigens. Thus, chronic alcohol consumption interferes with normal functioning of both adaptive and innate immune response, which increases sensitivity to viral and bacterial infections, as well as the development of inflammatory reactions [71].

## 5. Conclusions

Despite the significant progress in the study of neurotrophin functions and their implication in alcohol consumption effects and chronic inflammation, many knowledge gaps still remain. For instance, the role of NT-4 in the development of alcoholism is currently unknown. Some answers could be obtained by measuring NT-4 levels in serum of alcohol-dependent patients or measure NT-4 expression under oxidative stress conditions. In addition, it is clear that neurotrophins are involved in both the functioning of the immune system and the pathogenesis of alcoholism. New aspects of these interactions and intersections are constantly reported. Currently, there is an extensive discussion about which of the neurotrophin isoforms exhibit greater biological activity, as well as whether the Trk/p75NTR/NGF triple complex is formed. It is not known how exactly pro-BDNF can switch signals from apoptosis to cell survival, and what contributes to the shift of this equilibrium. Finally, the molecular mechanisms of analgesia due to prolonged alcohol withdrawal are also not entirely clear, and further research on this topic is needed.

## Figures and Tables

**Table 1:** Potential role of neurotrophins in alcohol addiction and immune system regulation.

Neurotrophin	Effect	Possible mechanism	Reference
**Alcohol consumption**			
NGF	Increased level of NGF; possible protective function	Prevents alcohol-induced neuronal death in rats	[43,44,45,46,47]
BDNF	Pro-BDNF increased, while mBDNF decreased; controls alcohol consumption	Polymorphisms in BDNF gene associated with the risk of alcoholism development; promotes survival of neurons	[48,49,50,51]
NT-4	Not clear yet	Possible role in alcohol-induced oxidative stress	[52]
**Immune system regulation**			
NGF	Pro-inflammatory	Promotes survival of immune cells	[4,55]
BDNF	Produced by activated immune cells; analgesic effect	Promotes survival of neurons	[56,57]
